# The Impact of Genetic and Non-Genetic Factors on Warfarin Dose Prediction in MENA Region: A Systematic Review

**DOI:** 10.1371/journal.pone.0168732

**Published:** 2016-12-19

**Authors:** Loulia Akram Bader, Hazem Elewa

**Affiliations:** College of Pharmacy, Qatar University, Doha, Qatar; Universita degli Studi di Roma Tor Vergata, ITALY

## Abstract

**Background:**

Warfarin is the most commonly used oral anticoagulant for the treatment and prevention of thromboembolic disorders. Pharmacogenomics studies have shown that variants in *CYP2C9* and *VKORC1* genes are strongly and consistently associated with warfarin dose variability. Although different populations from the Middle East and North Africa (MENA) region may share the same ancestry, it is still unclear how they compare in the genetic and non-genetic factors affecting their warfarin dosing.

**Objective:**

To explore the prevalence of *CYP2C9* and *VKORC1* variants in MENA, and the effect of these variants along with other non-genetic factors in predicting warfarin dose.

**Methods:**

In this systematic review, we included observational cross sectional and cohort studies that enrolled patients on stable warfarin dose and had the genetics and non-genetics factors associated with mean warfarin dose as the primary outcome. We searched PubMed, Medline, Scopus, PharmGKB, PHGKB, Google scholar and reference lists of relevant reviews.

**Results:**

We identified 17 studies in eight different populations: Iranian, Israeli, Egyptian, Lebanese, Omani, Kuwaiti, Sudanese and Turkish. Most common genetic variant in all populations was the *VKORC1 (-1639G>A)*, with a minor allele frequency ranging from 30% in Egyptians and up to 52% and 56% in Lebanese and Iranian, respectively. Variants in the *CYP2C9* were less common, with the highest MAF for CYP2C9*2 among Iranians (27%). Variants in the *VKORC1* and *CYP2C9* were the most significant predictors of warfarin dose in all populations. Along with other genetic and non-genetic factors, they explained up to 63% of the dose variability in Omani and Israeli patients.

**Conclusion:**

Variants of *VKORC1* and *CYP2C9* are the strongest predictors of warfarin dose variability among the different populations from MENA. Although many of those populations share the same ancestry and are similar in their warfarin dose predictors, a population specific dosing algorithm is needed for the prospective estimation of warfarin dose.

## Introduction

Warfarin is the most widely used oral anticoagulant for the treatment and prevention of thromboembolic manifestations associated with atrial fibrillation, prosthetic heart valves, orthopedic surgery, and history of vascular thrombosis [1, 2]. For example, in the United States alone, around 30 million warfarin prescriptions are dispensed annually, and in the year of 2010, total direct expenditures on warfarin were estimated to be around $600 million [2, 3]. Warfarin is a vitamin K antagonist that mediates its anticoagulant effect through preventing the activation of several vitamin K dependent coagulation factors including:- factors II, VII, IX, and X [4, 5]. Because of its narrow therapeutic index and wide interpatient variability, warfarin therapy requires close monitoring and repeated dose adjustments to achieve and maintain therapeutic anticoagulation effect [6–8]. Studies have repeatedly shown that genetic and non-genetic factors are contributing to the warfarin dose variability [9–11]. The most important genes consistently affecting warfarin dose among different populations are the *CYP2C9*- a gene coding for cytochrome P450 2C9 enzyme which metabolizes the more potent *S* enantiomer of warfarin, and *VKORC1*- a gene coding for the vitamin K epoxide reductase which is an enzyme inhibited by warfarin [9, 10]. Mutations in the gene coding for the CYP4F2, a metabolizing enzyme for vitamin K, have also been shown to contribute to warfarin dose variability but to a lesser extent, and its effect was not consistent among all populations [12, 13]. The Clarification of Optimal Anticoagulation through Genetics (COAG) and the European Pharmacogenetics of Anticoagulant Therapy (EU-PACT) were the largest randomized controlled trials designed to assess the utility of genotype-guided dosing [14, 15]. Despite the negative results from the COAG trial indicating no benefit of genetic-guided dosing, compared to clinical dosing, it showed that the percent time in therapeutic range (PTTR) was significantly lower in blacks in the genetic-guided arm compared to the clinical dosing arm [14]. This is probably due to the fact that blacks may have other less-common genetic variants affecting their warfarin dose that were not well-represented in the genetic-algorithm used in the COAG trial [16]. The EU-PACT study on the other hand, compared pharmacogenetics-based doing versus fixed-dose strategy and was performed in a predominantly white population from Europe [15]. In this study, mean PTTR at 3 months was significantly higher in the genotype-guided group than in the control group [15].

Due to its strategic location and its resources, the Middle East and North Africa (MENA) region was always left at conflict, with different civilizations migrating in and out of its countries. That in turn have put populations of MENA through genetic admixture and racial mixing, creating heavily admixed populations with Asian, Caucasian, Arab and African ancestries. Numerous studies in different countries of MENA have been conducted to estimate the frequencies of the genetic mutations associated with warfarin dosing and its ability along with other non-genetic factors to predict warfarin dose. However, it is still not very clear to what extent the results of these studies are comparable and whether all or some of these results should be pooled together to come-up with a more accurate algorithm. As such, we felt that a summary of the evidence on warfarin pharmacogenetics in this region is needed in-order to identify the gaps and provide future directions. Our goal was to systematically review studies from the MENA region that estimate the impact of genetic and non-genetic factors on warfarin dose requirements.

Study objectives: to systematically review all observational studies that have explored the effect of both genetic and non-genetic factors on warfarin dose variability in the MENA region, estimate the prevalence of the studied genetic variations that are associated with warfarin dose variability, and compare the performance of different genetic-based algorithms in warfarin dose prediction.

## Methods

### Search Strategy

A systematic comprehensive search for observational studies was applied with no date or language restrictions. The search started on February 2016 and was completed by the end of March 2016.

#### Database search

PubMed, Scopus, MedLine, PharmGKB (Pharmacogenomics Knowledge Base), and PHGKB (Public Health Genomics Knowledge Base) were searched using different terms with the appropriate Booleans. Below is an example of the search terms and Boolean used to search PubMed. Similar terms were used to search other databases, for Scopus and MedLine results were refined by country.

- Combination #1: “warfarin” AND “dose prediction” AND “genetic polymorphism” (In title and abstract).
- Combination #2: “warfarin” AND “dosing” AND “genetic mutations” (In title and abstract).
- Combination #3: “warfarin” AND “genotype guided” AND “algorithm” (ALL fields).
- Combination #4: “warfarin” AND “polymorphism” AND “dose prediction”.
- Combination #5: #1 OR #2 OR #3 OR #4.

#### Other resources

For grey literature, Google Scholar was searched without any language or date limits. Moreover, reference list of all kind of relevant review articles retrieved during our search, were hand searched to identify any potential study.

### Study Types

Observational cross sectional or cohort studies were included. Randomized controlled studies, case control, case series, and qualitative studies were excluded from this review.

### Participants

Adult patients from MENA region, who have been on warfarin for a sufficient amount of time to have a stable dose and their International Normalized Ratio (INR) within the therapeutic range.

### Outcome Measure

Included studies were required to have mean warfarin dose as their primary outcome and to have estimated the variability in the dose explained by genetic and non-genetic factors.

### Eligibility Criteria

Studies were considered eligible for this review if they have studied populations of the MENA region, have explored the effect of both genetic and non-genetic factors on warfarin dose, and have developed a dosing model.

### Study Selection

Potentially relevant studies were screened first by title and abstract to exclude any irrelevant studies. Then studies were screened by text to exclude any study that does not apply the inclusion criteria. Both former steps were carried by two reviewers (HE and LB), independently. Whenever there was a disagreement, it was resolved by discussion.

### Data Extraction and Management

Data were extracted with a pre-specified and piloted extraction tool that was prepared and reviewed by the authors. Abstracted data included: author and year of publication, population studied, sample size, genetic and non-genetic factors explored, mean warfarin dose, mean INR, and main findings.

### Quality Assessment

Quality assessment tool for observational cohort and cross sectional studies, adopted from the NIH, National Heart, Lung, and Blood Institute [17] was used to assess the quality of all included studies.

### Data Synthesis

All extracted data were summarized and presented descriptively.

## Results

### Study Selection

**Fig 1:**
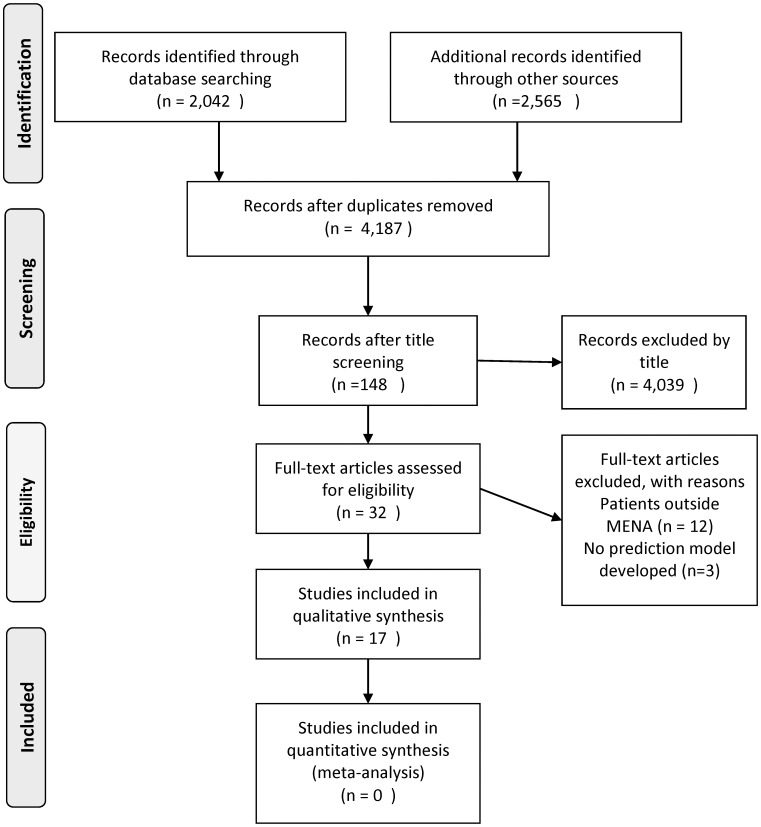
PRISMA Flow Chart of Included Studies.

A total of 17 studies were included in this review, including 8 different populations. Fig 1 shows a flow chart of the included studies. Out of 32 studies screened for eligibility, 12 studies were excluded for not targeting patients from the MENA region [7, 9, 10, 18–26] and 3 studies were excluded [27–29] as they did not develop a dosing model. We identified studies from 8 different countries including: Egypt, Iran, Lebanon, Turkey, Sudan, Oman, Kuwait, and two studies in occupied Palestine on Israeli population. Characteristics of all studies are shown in Table 1.

**Table 1:** study Characteristics.

Study	Population	Sample size	Genetic factors explored	Non-genetic factors explored	Mean warfarin dose
*Shahin et. al. 2011, 2013* [12, 30]	Egyptians	207	*CYP2C9*(*2, *3, *4, *5, *8),*VKORC1* (3673G>A, Asp36Tyr), *APOE*, *CYP4F2*(Val433Met), *CALU*	Sex, age, BSA, use of aspirin, indication for warfarin, concomitant disease & smoking status	36.8 ± 17.9 mg/wk
*Bazan et. al. 2014* [31]	Egyptians	63	*CYP2C9*(*2, *3), *VKORC1*	Age & smoking status	7.3 ± 5.2 (1–30) mg/day
*Ghozlan et. al. 2015* [32]	Egyptians with ACS	80	*CYP2C9*, *VKORC1*	Age & height	4.8 ± 1.96 (2–10) mg/day
*Issac et. al. 2014 & Ekladious et. al. 2013* [33, 34]	Egyptian	84 (50 for model, 34 for validation)	*CYP2C9*(1075A>C), *VKORC1*(1173C>T), *R1*, (C3435T), *EPHX1*(H139R), *PZ*(A-13G)	Age & gender	
*Namazi et. al. 2010* [35]	Iranian	100 total (100 CYP2C9, 99 CYP2C19, 81 VKORC1) 55 for the model	*CYP2C9* (*2, *3), *VKORC1* (-1639G>A), *CYP2C19* (*2, *3)	Gender, age, BSA, weight & height	7.3 ± 5.2 (1–30) mg/day
*Loebstein et. al. 2005* [36, 37]	Israeli	100	*GGCX*, *CALU*, *VKORC1*, *EPHX1*, *CYP2C9* (*2, *3)	Age, weight, concurrent medication & total vit.K plasma concentration	5.7 ± 3.3 (1.1–20) mg/day
*Alrashid et. al. 2016* [38]	Kuwaiti	108	*CYP2C9*, *VKORC1*	Sex & BMI, age were adjusted for in the model but not included as predictors	4.7 ± 2.7 mg/day
*Esmerian et. al. 2011* [39]	Lebanese	43	CYP2C9 (*2, *3), *VKORC1* (-1639G>A, 1173 C>T)	N/A	31 ± 14 mg/wk
*Pathare et. al. 2012* [40]	Omani	212 (142 in the model derivation cohort, 70 for validation)	*CYP2C9* (*2, *3), CYP4F2 *3, *VKORC1* (3673G>A, 5808T>G, 6009C>T, 6484C>T, 9041G>A)	Simvastatin, amiodarone, hypertension, diabetes, atrial fibrillation, deep vein thrombosis, mechanical valve, age, weight & gender	4.75 (3–5.5) mg/day
*Shrif et. al. 2011* [41]	Sudanese	203 patients 180 healthy volunteers	*CYP2C9* (*2, *3, *5, *6, *8, *9, *11), *VKORC1* (20 tagSNPs)	Body weight, concurrent medication, target INR, body surface area, height, age, indication for warfarin treatment & gender	5.58 ± 2.48 (1.5–22.5) mg/day
*Özer et. al. 2013* [42]	Turkish	107	*CYP2C9* (*1,*2), *VKORC1*(-1639G>A, 1173C>T), *CYP4F2*, *EPHX1*	Age, height, weight, No. of cigarettes & daily consumed tea and green vegetables	5.16 ± 1.95 (1.43–10) mg/day
*Ozgon et. al. 2008* [43]	Turkish	205	*CYP2C9* (*2,*3,*4,*5), *VKORC1* (-1639G>A)	Age & non-indication of VT	34.2 ± 16.78 (6.25–125) mg/wk
*Yildirim et. al. 2014* [44]	Turkish	101	*CYP2C9* (*2,*3), *VKORC1*(-1639G>A), *factor VII* (-401G>T)	Age, BMI & INR	4.07 ± 1.6(1.13–7.86) mg/day
*Ozer et. al. 2010* [45]	Turkish	100	*CYP2C9*(*2,*3), *VKORC1*(-1639G>A)	Age & BSA	4.11 (1.16–9.33) mg/day

After assessing for quality, most studies were found to be ranging from fair to good quality. However, the study by Alrashid et al [38] was considered of poor quality as there was no reporting of the r^2^ value for the dosing model. The sample-size used in some of the studies was less than 100, which is relatively low and may have affected the power of their conclusions [31, 32, 34, 39]. Testing for the Hardy-Weinberg equilibrium was reported in all studies except those by Yildirim et al [44] and Ghozlan et al [32].

Targeted populations were required to be on stable warfarin dose as part of the inclusion criteria. Due to the variability in the definition of stable warfarin dose among studies, we reported the different definitions used by authors of each included study (Table 2). Overall, having an INR within therapeutic range for three consecutive visits while on the same warfarin dose was considered “stable warfarin dose” description by almost all studies.

**Table 2:** Stable Warfarin Dose Definition Variation Among Studies.

Studies	Definition
*Shrif et. al. 2013* [41]	Mean of the daily warfarin doses at which INR measurements were within target therapeutic levels for three consecutive clinic visits over more than 3 months
*Namazi et. al. 2010* [35]	Warfarin dose that was constant for ≥ 3 consecutive visits over a minimum period of 3 months with INR value variation ≤ 15%
*Esmerian et. al. 2011* [39]	Therapy for at least 2 months, same weekly dose of warfarin over the past 3 INR examinations, patients considered adequately coagulated if the INR value at recruitment fell within a range of 1.7–4
*Pathare et. al. 2012* [40]	A patient was considered to have a stable INR when his/her INR was between 2 and 3 on at least 3 consecutive assessments, 3 months after initiating the therapy
*Alrashid et. al. 2016* [38]	Patients were defined as those whose warfarin dose requirement has remained constant for at least three consecutive clinic visits, at which point the INR was within the therapeutic range
*Bazan et. al. 2014* [31]	Patients having a stable warfarin dose requirement for at least 3 consecutive times with dose titration to an INR target range of 2–3.5
*Shahin et. al. 2011, 2013* [12]	A dose that did not vary by more than 10% between clinic visit, for three consecutive visits, occurring over a minimum time-period of 2 months
*Ghozlan et. al. 2015* [32]	Steady-state dose that leads to stable anticoagulation levels in three consecutive clinic visits for which the INR measurements are within the range of 2–3
*Yildirim et. el. 2014* [44]	Not clearly defined
*Özer et. al. 2013* [42]	Patients were included if they had been on therapy for > 4 months and their last three INR measurements were within therapeutic range for the same mean daily dose
*Ozgon et. al. 2008* [43]	Patients were included if they had been on therapy >2 months and their last three INR measurements were considered stable by their doctors, whether or not they correspond to their target INR
*Loebstein et. al. 2005* [37]	Having therapeutic INR over 4 consecutive visit and receiving the same daily dose of warfarin before sample collection on index visit
*Issac et. al. 2014 & Ekladious et. al. 2013* [33, 34]	Having at least three consecutive INRs in the therapeutic (2–3) range for the same daily maintenance dose after at least 3 months of therapy
*Ozer et. al. 2010* [45]	Three consecutive clinic visits for which INR measurements were within therapeutic range for the same daily dose

### Prevalence of the studied genetic variants

In-order to estimate the prevalence of different genetic variants we reported the minor allele frequency (MAF) of these variants. For the most common genetic variants in *VKORC1* and *CYP2C9*, MAF’s are presented in Table 3. Variants in the *VKORC1* gene had the highest frequencies among all populations followed by the *2 variant of *CYP2C9* gene and *3, with *2 being relatively higher in most of the populations.

**Table 3:** Minor Allele Frequency (MAF) of Most Common Genetic Variants.

Population	Gene	*VKORC1*	*CYP2C9*	
	Variant	-1639 G>A (rs9923231)*	*2(rs179853)	*3(rs1057910)
Egyptians [12, 30]		46.2%	11.7	9.2%
Egyptians [34]		72.05%	N/A	10.7%
Egyptians [31]		51%	7%	9.6%
Egyptians [32]		30%	8%	4.3%
Iranian [35]		56%	27%	9%
Israeli [36]		N/A	12.5%	11%
Kuwaiti [38]		40%	14%	5%
Lebanese [39]		52%	15%	7%
Omani [40]		35%	6%	6%
Sudanese [41]		37%	5%	0%
Turkish [43]		50%	13%	10%
Turkish [42]		49%	N/A	N/A
Turkish [45]		40%	13%	15%
Turkish [44]		51%	17%	27%

* This rs ID refers to *VKORC1* (-1639G>A) and *VKORC1*(3673G>A)

Other less common genetic variants have been explored in Egyptians, Israeli, Omani, and Turkish populations. These include variants in the following genes: *CYP4F2*, *EPHX1*, *GGCX1*, *MDR1*, *factor VII*, *APOE*, *CALU* and *PZ*. The MAF for the *CYP4F2* (rs2108622) was 40% among Turkish [42], 30% in Omani [40] and 42% in Egyptians [12]. The *EPHX1* encodes for a putative subunit of the vitamin K epoxide reductase complex called: microsomal epoxide hydrolase 1 [10]. The MAF of *EPHX1* (rs2292566) was 16% and 26.19% in Turkish and Egyptians, respectively [33, 42], while another variant the (rs1051740) was 25% in Israeli [36, 37]. The multidrug resistance gene (*MDR1* C3435T) and *Protein Z* A-13G were only studied in Egyptians and their MAF were 42.86% and 0%, respectively [33]. Other gene variants that are associated with warfarin dose variability are the *APOE* and *CALU* [10]. The rs429358 and rs7412 variants of the *APOE* gene had a MAF of 6.7% and 7.4%, in Egyptians, respectively [12]. On the other hand, the rs339097 variant of the *CALU* gene was less common in Egyptians than the *APOE* variants with a MAF of 2.3% [12]. Other rare genetic variants that have been explored are the *Factor VII* (-401G>A) which had a MAF of 35% in Turkish [42], and the *GGCX* (rs699664) which had a MAF of 29.5% in Israeli [36].

### Significant Predictors of Warfarin Dose

Using univariate and multivariate linear regression, authors of all studies were able to identify the most significant predictors of warfarin dose and the most significant dosing model which explained the highest percent of the dose variability. Table 4 shows the significant predictors in the different populations and to what extent were they able to predict warfarin dose.

**Table 4:** Most Significant Predictors and % Variability Explained.

Population	Significant Genetic Predictors	Significant Non-Genetic Predictors	Variability explained by the model
**Egyptians** [12, 30]	V*KORC1* (-1639G>A) & (Asp36Tyr) *CYP2C9*(*2,*3,*4,*5,*8) APOEε2	Age Pulmonary embolism Smoking status	36.5%
**Egyptians** [32]	VKORC1(-1639G>A) CYP2C9 *2 & *3	Age Height	30.6%
**Egyptians** [31]	VKORC1 (-1639G>A) CYP2C9*3	Age Smoking status	43.4%
**Egyptians** [33, 34]	*VKORC1*(1173 C>T) *MDR1* (p = 0.055)	Age	20.9%
**Iranian** [35]	*VKORC1* (-1639G>A) *CYP2C9* *2 & *3	Age Sex Height	41.3%
**Israeli** [37]	*VKORC1* (1542G>C) *CYP2C9* *2 & *3	Age Weight	63%
**Kuwaiti** [38]	*VKORC1* (-1639G>A) *CYP2C9* *2 & *3	Age Body Mass Index	N/A
**Lebanese** [39]	*VKORC1* (-1639G>A) *CYP2C9* *2 & *3	N/A	33.9%
**Omani** [40]	*VKORC1* 3673(GA & AA) *CYP2C9* (*2/*3, *3/*3)	Atrial fibrillation	63%
**Sudanese** [41]	*VKORC1*(rs8050894,rs7199949, rs7294), *CYP2C9* (*2,*5,*6,*11)	Concurrent medication Indication for warfarin	36.75
**Turkish** [43]	*VKORC1* (3673 G>A) *CYP2C9* *2, *3, & *4	Age Non-indication of VTE	43%
**Turkish** [45]	*VKORC1*(-1639 G>A) *CYP2C9* *2 & *3	Age Body surface area	60.4%
**Turkish** [42]	*VKORC1*(-1639G>A) *CYP2C9* *2 &*3 *CYP4F2* (rs2108622)	Age	39.3%
**Turkish** [44]	*VKORC1* (-1639G>A)		18.2%

## Discussion

### Summary of Evidence

The term MENA refers to countries from Morocco to Iran and down to Sudan. Studies of maternally inherited mitochondrial DNA have suggested that all modern populations, including those of MENA [46–48], have originated through a single wave from Sub Saharan Africa and populated other parts of the world [49]. Later-on, several historical events took place and facilitated genetic migration like the early Islamic conquests, the Ottoman Empire expansion and the continuous migrations in and out of the Arabian Peninsula and Europe. Other examples include the silk and spice road, which used to connect China with Europe through the Middle East [50]. Countries like Egypt and Oman, their strategic crossroad position between Africa and Eurasia have left them with high intrapopulation diversity [46]. On the other hand, other countries like Qatar and Yemen have the highest rates of consanguinity, 44.7% and 54%, respectively, leaving them with less genetic variability [51]. All of the aforementioned reasons have made these countries of particular interest in genetic studies.

Pharmacogenetics studies have shown that variants of *VKORC1* and *CYP2C9* are strongly associated with warfarin dose. *VKORC1*(-1639G>A) alone can explain up to 40% of the dose variation in some populations [52]. Along with these genetic predictors, other genetic factors and non-genetic factors such as age, weight, gender, race, ethnicity, concurrent medication, and comorbidities can boost the preemptive estimation of warfarin dosing to more than 50% [10].

The fact that *VKORC1* variants are the most significant predictors of warfarin dose followed by those of *CYP2C9*, was consistent across all studies included in this systematic review. However, the frequency of these genetic variations and the extent of their effect on dose variability varied from population to another. Similarities were seen between Sudanese and African Americans in that CYP2C9*5, *6, and *11 were better predictors of warfarin dose than the *2 and *3 variants [53]. Moreover, the variability explained by variants of *VKORC1* (rs9934438, rs9923231, rs8050894) were comparable to those of African Americans [1, 53]. CYP2C9*8 (rs9332094) is another rare variant that affects around 12% of African Americans and has been associated with reduced *S*-warfarin clearance [54]. This SNP was only investigated in two populations of MENA; Egyptians and Sudanese [12, 41]. Despite some of the similarities found between these two populations and African Americans, the CYP2C9*8 did not shown any significant association with warfarin dose requirements in Egyptians and Sudanese as it did in African Americans. This might be in part due the fact that this variant was not very common in these populations, it showed a MAF of 0.008 and 0.01 in Egyptians and Sudanese, respectively [12, 41]. On the other hand, the frequencies of *CYP2C9* *2 and *3 and *VKORC1* (3673G>A) reported in Turkish [43] were closer to Caucasians than to Asians and African Americans [1].

Variants of the *VKORC1* gene were the most prevalent among all populations. Great discrepancy was seen in the frequency of the *VKORC1* in Egyptians, it ranged from 30% in the study be Ghozlan et. al and up to 72% in the study by Ekladious et. al. [32]. Such discrepancy could be in part due to the small sample sizes used in the different studies concerning the Egyptian population, in particular the studies by Ghozlan, Ekladious, and Bazan and their colleagues [31, 32, 34]. Moreover, the Egyptian population is a heavily admixed population with different ethnic origins [12] which may have led to different frequencies depending on the ethnicity of the studied population. However, none of these studies have reported the different ethnic origins of their studied population and whether it was a contributing factor or not. Carriers of the -1639G>A variant required lower warfarin dose compared to the wild type in all studies. Shrif and colleagues were able to identify four different SNPs downstream from the *VKORC1* gene (rs7199949, rs11865038, rs11864839, and rs750952). These SNPs were found to be located in newly identified gene which is only 2 kb downstream from the *VKORC1* gene in the same reverse strand named: *POL3S* (polyserase 3S) [41]. Amino acid sequences of the POL3S are highly conserved among humans, chimpanzees, dogs, cows, and mice [55]. While function of the encoded protein is still unknown in-vivo, it has shown to degrade the alpha chain of fibrinogen as well as pro-urokinase type plasminogen in vitro, indicating its potential role in the coagulation process [55]. Out of the identified SNPs in the *POL3S*, rs7199949 (p.Pro406Ala) proved to be a significant predictor of warfarin dose and its addition to the regression model explained 7% of the variability [41].

Variants in the *CYP2C9* were less common than those of the *VKORC1* in all populations with the *2 variant being slightly more common than the *3 variant in majority of cases. The *2 variant was highest among Iranian (27%) [35], while the *3 variant was highest among Turkish (27%) and it was not detected in Sudanese [41, 44]. MAF of *2 and *3 were closely similar among Egyptians, Israeli, and Turkish [30, 36, 43]. Both *2 and *3 variants were associated with lower warfarin dose requirements in all populations.

The *VKORC1* (-1639G>A) variant was the most significant predictor of warfarin dose in all population. This variant alone was able to explain 45% of the dose variability in Omani [40]. The *VKORC1* Asp36Tyr was significantly associated with higher warfarin doses in Egyptians and when added to Shahin et al. dosing model it further explained the variability by only 2% [30].

*CYP4F2* is the gene coding for the cytochrome P450 4F2 enzyme, which is a vitamin K oxidase. The V433M allele is associated with reduced metabolism of vitamin K1 and higher warfarin doses [56]. Caldwell et al. has shown that *CYP4F2* V433M (rs2108622) variant has an impact on warfarin dose and that carriers of at least one minor allele required 4–12% higher doses than those with homozygous wild type genotype [57]. Only 3 studies in three different populations of MENA -Egyptian, Omani, and Turkish- have investigated the association between warfarin dose and that gene variant. These studies have shown no significant association between *CYP4F2* gene variant and warfarin dose in Egyptians neither in Omani, despite its high frequency [12, 40]. A significant association between *CYP4F2* gene variant and warfarin dose was among Turkish by Özer et. al [42]. Furthermore, Turkish carriers of the variant allele required higher warfarin doses than those with the wildtype. The *APOE* gene encodes for an apolipoprotein that is involved in the hepatic clearance of vitamin K [10]. In the Egyptian cohort studied by Shahin. et. al., carriers of the *APOE ε2* gene variant required lower warfarin dose compared to the wild type [12]. Moreover, that variant explained 2.53% of the dose variability. No association was found between *MDR1* C3435T, *EPHX1* H139R, *GGCX* and *protein Z* A-13G gene variations and warfarin dose in populations of MENA. However, when combined, these genetic variants were able to explain 3% of the dose variability in Egyptians [33]. Calumenin (CALU) is a regulator of vitamin K epoxide reductase (VKOR) as identified in studies of warfarin resistance rat and it have been linked to warfarin sensitivity [58]. Shahin et al. was able to confirm the association of *CALU* rs339097 gene polymorphism with higher doses of warfarin, however, when added to the regression model it failed to show any significance [12]. The authors reported that failing to show significance may have been due to lack of power, since only nine variant carriers were found in their cohort. In African Americans, it was shown, that for every *CALU* rs339097 variant allele warfarin dose increases by almost 11% [58]. Factor VII is a vitamin K dependent factor, *factor VII* -401G>T variant was able to predict only 2.2% of warfarin dose variability among Turkish, even though it had an MAF of 35% [44]. Carriers of the minor allele required lower warfarin dose as opposed to the carriers of the major allele [44].

After developing their dosing algorithm, Pathare et al. compared the performance of their algorithm with that of the International Warfarin Pharmacogenetics Consortium (IWPC) in predicting warfarin dose in their Omani cohort [40]. The IWPC algorithm was able to explain only 33.6% of the variability, while Pathare et al. algorithm explained 63% of the variability [40]. Moreover, warfarin dose predicted by the IWPC algorithm was 13% higher than the patients’ actual dose [40]. Even though the IWPC algorithm has been developed and validated using data from different racial groups including Asians, Blacks, and Whites across four different countries, and even though the Omani population comes from Asian, African and Caucasian ancestries, the IWPC algorithm did not perform well in the Omani population [40]. Such variation in the algorithm performance could be owed to the fluctuation in racial definitions, clinical variations, social/lifestyle contexts, population migration and historical perspectives [59]. Such observation highlights the need for dosing algorithm that are more population-tailored.

Beside the genetic variants other clinical and demographic variables were shown to be significantly associated with warfarin dose. Age, weight, gender, body mass index and body surface area were the most significant predictors of warfarin dose and added up to the variability explained.

## Limitations

Some of the limitations of this review were having only two reviewers carrying the whole process independently which may have led us to overlook some important findings or observations. Another limitation is that we only considered mean warfarin dose as our primary outcome, while other outcomes like Time to Therapeutic Range (TTR) and risk of bleeding might be predicted by genetic factors. Furthermore, SNPs like the *GGCX*, *Factor VII*, *CALU*, *APOE*, *MDR1*, *EPHX1*, and *PZ* were investigated in limited number of studies, which made it unfeasible for us to compare their effect on warfarin dose between different populations of MENA.

### Conclusions and Future Directions

*VKORC1 & CYP2C9* were the most useful variants in predicting warfarin dose. Despite the shared significant predictors in all dosing models, their performance still varied largely between populations. As such, micro-geographically defined population specific dosing algorithms are needed. Warfarin pharmacogenetics have been explored in limited number of populations in MENA region. Thus, more pharmacogenetics studies on other populations are needed to further explain the impact of genetic factors on warfarin dose in the region. Future clinical utility and cost effectiveness studies will confirm if warfarin genetic dosing strategy should replace clinical dosing.
